# Artificial intelligence in glycemic management for diabetes: Applications, opportunities and challenges

**DOI:** 10.1515/jtim-2025-0039

**Published:** 2025-08-12

**Authors:** Zhen Ying, Xiaoying Li, Ying Chen

**Affiliations:** Ministry of Education Key Laboratory of Metabolism and Molecular Medicine, Department of Endocrinology and Metabolism, Zhongshan Hospital, Fudan University, Shanghai, China

## Background

Diabetes mellitus, affecting 10% of the global population, stands as one of the most prevalent chronic diseases worldwide. Over half of these patients experience inadequate glycemic control, highlighting the shortcomings of conventional management methods. Given the economic and personal impact of diabetes, including decreased productivity, higher healthcare costs, severe complications and shortened lifespan, there is a pressing need for more efficient and cost-effective glycemic management tools. Nowadays, artificial intelligence (AI), which harnesses the power of big data and sophisticated algorithms, has offered promising solutions to improve patient outcomes and reduce the burden on healthcare providers. In this context, we demonstrate the current applications of AI in glycemic management and discuss the opportunities and challenges that AI faces in diabetes management.

## Applications of AI in glucose monitoring and prediction

Glucose monitoring plays a pivotal role in diabetes management, encompassing hemoglobin A1C (A1C) measurement, blood glucose monitoring (BGM) by capillary (finger-stick) devices, and continuous (interstitial) glucose monitoring (CGM). AI has been employed in glucose monitoring and prediction to provide an early indicator of glycemic control status and detection of adverse events in patients with diabetes.

A1C, which reflects average glucose over the last 2–3 months, has been validated as a measure of both long-term glycemic control and risk for the development of complications. Several studies have constructed AI-driven HbA1c predictive models to forecast individual treatment outcomes and reflect the prognosis of diabetes, showing the potential of AI methods in long-term diabetes management.^[1]^

Although A1C is significant, it does not adequately reflect glycemic variability and patterns, making it insufficient for patients requiring immediate monitoring and treatment adjustments. In contrast, BGM and CGM provide more detailed and timely glucose response data. Capillary blood glucose monitoring is widely used to assess glucose levels and guide both self-and clinician-directed management. CGM allows continuous, real-time monitoring of interstitial glucose, offering insights into glycemic excursions, daily profiles, and variability. AI algorithms based on BGM could predict future BG and adverse events, providing early warnings of hyperglycemia or hypoglycemia.^[2]^ Notably, ongoing improvements in the accuracy and convenience of CGM devices have prompted the increasing adoption of this technology. Several studies have utilized the combined data from CGM and other wearable devices like physical activity monitors and meal tracking to understand the precise glucose change of patients deeply and further support personalized treatment.^[3]^

## AI used in optimizing diabetes management

While traditional diabetes management confronts challenges such as poor patient adherence, lack of personalized care, and limited medical resources, there are increasing applications of AI in diabetes education, lifestyle guidance, and pharmacologic therapy, offering a promising solution to provide optimal diabetes treatments.

### Diabetes education and lifestyle guidance

Diabetes education is the foundation of effective diabetes prevention and care, helping people with diabetes acquire the knowledge, skills, and behaviors necessary to perfect self-control of diabetes and promote quality of life. Confronted with the shortage of resources required for needed educational interventions, AI solutions can potentially expand care delivery by providing professional knowledge and personalized lifestyle guidance. Knowledge graph (KG), a structured representation of knowledge that captures information in a machine-readable format, has been employed to conduct comprehensive and professional datasets to support further medical information retrieval, health care education, and individual intelligent question and answering (QA). Smart conversational agents, especially large language models have shown potential as tools for direct patient engagement and education through human-like responses.^[4]^ Meanwhile, AI-powered health care platforms could reinforce physical-activity adherence, reduce the burden of diet tracking, facilitate personalized feedback, and foster more effective diabetes self-management.^[5]^

### Anti-diabetic agents

Selecting target-specific anti-diabetic drug therapy is crucial but challenging in patients with diabetes (particularly T2D) due to complicated prescriptions and the individual heterogeneity. A review of studies leveraging AI to predict the use of anti-diabetic agents highlights the promise of precision medicine, tailored to individual patient traits using AI.^[6]^ Meanwhile, recent research showed the responses generated by the commercially available large language model GPT-4 were superficially reasonable and personalized to align with clinical guidelines, but differed systematically from endocrinologists’ responses in clinically meaningful ways. The results indicate that AI tools have potential for optimizing drug treatment plans for people with complex diabetes, but further validation through randomized controlled trials is necessary.^[7]^ In addition, due to the exhaustive medication guides, it’s hard for patients to retain the necessary information, which can result in hospitalizations and medication nonadherence. The advancements in AI also allow researchers to improve the design of AI- and human factor-based tool that supports patients’ medication information comprehension and potentially improves medication adherence.

### Insulin therapy

Optimizing insulin therapy is indispensable for T1DM and important for T2DM with disease progression. However, most patients receive suboptimal doses and do not achieve glycemic control due to inertia insulin titration, fear of hypoglycemia, and so on. Although a series of clinical guidelines on rational insulin use for patients with diabetes have been proposed by experts, they cannot fully take into consideration the heterogeneity of each patient in the real world. Several digital technologies have emerged to support the insulin management and glucose control, including basic bolus dose calculations, insulin titration algorithms based on AI, and autonomous continuous insulin infusion closed-loop systems.

Compared with the calculation method based on mathematical formulas, AI-driven insulin titration algorithms which consider the diverse and dynamic characteristics of patients are more suitable to address challenges in complex and fluid clinical settings. For example, we have built a reinforcement learning-based subcutaneous insulin titration for T2DM, which learns the optimal insulin regimen by analyzing glycemic state rewards through patient model interactions, and achieved superior performance compared to other models and standard clinical methods.^[8]^ In a randomized clinical trial of a voice-based conversational AI application that provided autonomous basal insulin management for adults with type 2 diabetes, participants in the AI group had significantly improved time to optimal insulin dose, insulin adherence, glycemic control, and diabetes-related emotional distress compared with those in the standard of care group.^[9]^

Automated insulin delivery systems, which mainly utilize proportional-integral-derivative (PID) and model predictive control (MPC) models, have successfully transitioned from the research bench to becoming the standard of care for people with type 1 diabetes. Meta-analyses show that hybrid closed-loop systems outperform non-automated systems with improvements in time spent in the target glucose range of approximately 8–12 percentage points, reduced time spent in hyperglycemia, reduced mean glucose, and either a reduction or no increase in time in hypoglycemia. Notably, while hybrid closed-loop systems are the mainstay of automated insulin delivery, fully automated insulin delivery solutions may become commercially available soon with the growing role of artificial intelligence in optimal glucose management.^[10]^

## Opportunities and challenges

**Figure 1:**
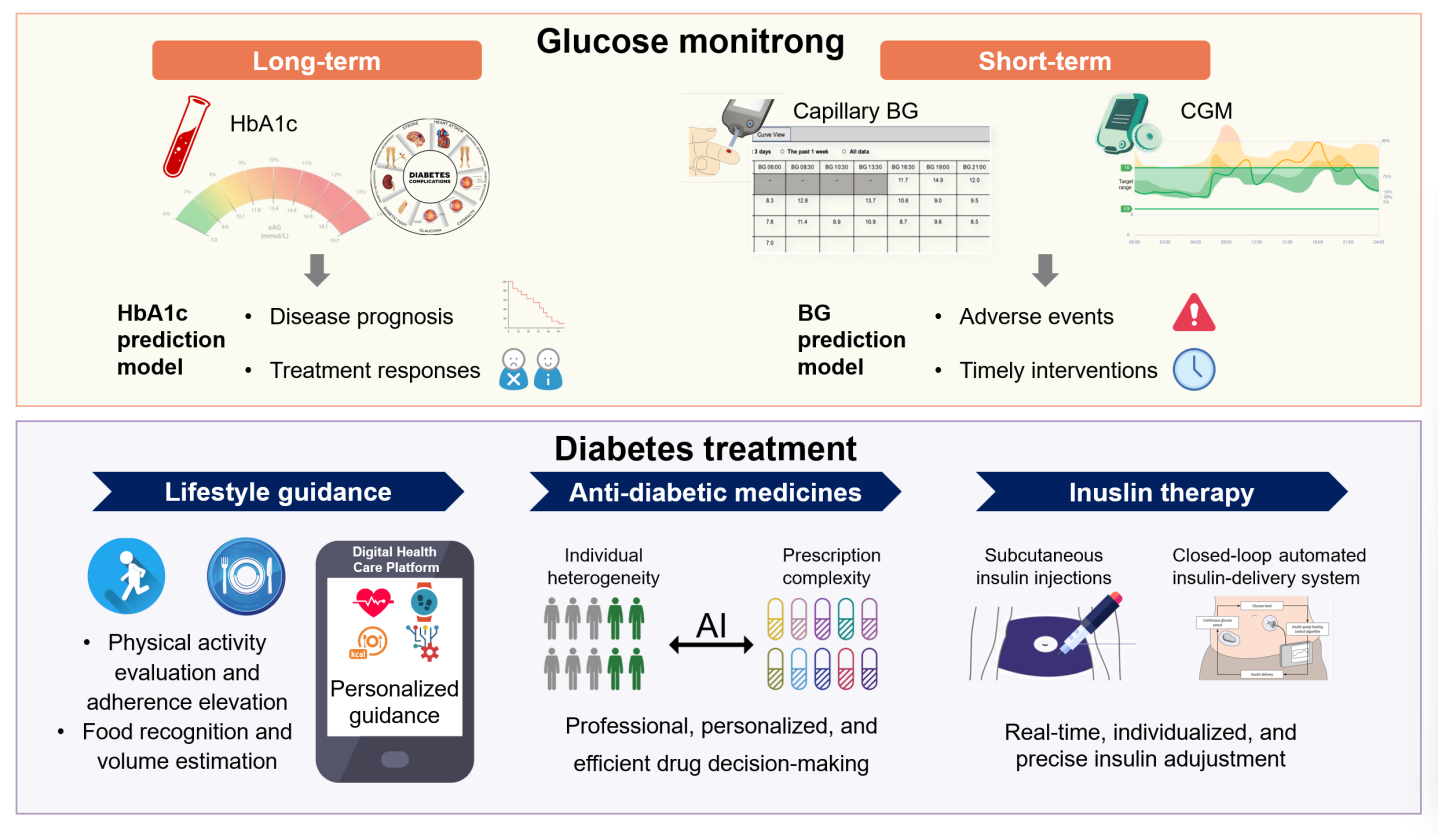
Applications of artificial intelligence (AI) in glycemic management for diabetes.

The advancement of AI technology enables more precise monitoring and prediction of short-term and long-term blood glucose levels and offers personalized lifestyle guidance, anti-diabetic medication, and insulin decision-making, providing new tools to improve glycemic control in diabetic patients and reduce the risk of complications (Figure 1).

**Figure 2:**
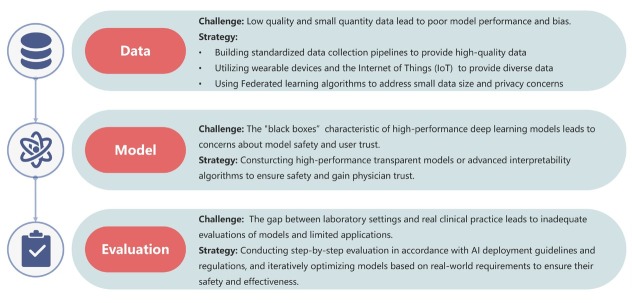
Challenges for artificial intelligence (AI) application in glycemic management and possible strategies.

However, AI applications in diabetes management still face challenges. First, low-quality or small datasets can result in inadequate model performance and biases, harming clinical practice. Collaborative efforts to establish standardized data collection platforms are crucial for overcoming this obstacle. Furthermore, the emergence of wearable devices and the Internet of Things (IoT) promises to yield a wider range of high-precision data. However, as the gathered data may encompass sensitive personal details, data privacy remains a paramount concern in healthcare AI. To safeguard privacy, regulations like the General Data Protection Regulation (GDPR) and California Consumer Privacy Act (CCPA) have been enacted. Simultaneously, innovative techniques such as federated learning (FL), blockchain technology, and generative adversarial networks are evolving to tackle this challenge. Second, while high-performance deep learning models are powerful tools, they often function as “black boxes”. Clinical practice requires interpretability from these models to ensure patient safety and foster trust among physicians. Hence, there is an urgent need for algorithmic innovation to construct high-performance transparent models or advanced interpretability algorithms to further expand the applicability of models. Third, a notable disparity exists between laboratory environments and actual clinical situations, and current evaluations of models are still inadequate. Consequently, during the implementation of AI, it is necessary to follow guidelines for step-by-step validation and iterate and optimize models based on real-world requirements to ensure their safety and effectiveness. Simultaneously, ethical questions arise regarding accountability in the event of AI errors, highlighting the importance of robust safety measures and unambiguous policies assigning responsibility in AI applications (Figure 2).

In conclusion, the advancement of AI is transforming diabetes management yet several challenges remain to be addressed. As physicians, we should not only embrace and refine these technologies to offer better healthcare services but also keep a cautious, patient-centered orientation to ensure safety.
